# Perception, Quality, and Accuracy of Sunscreen Content on TikTok: SkinMedia Cross-Sectional Content Analysis

**DOI:** 10.2196/70010

**Published:** 2025-12-01

**Authors:** Jaclyn Roland-McGowan, Kyra Diehl, Tayler Tobey, Autumn Shafer, Paige Clement, Oliver J Wisco, Alex G Ortega-Loayza, Sancy Leachman

**Affiliations:** 1 School of Medicine, Oregon Health and Science University, Portland, OR, United States; 2 Department of Dermatology, Oregon Health and Science University, 3303 SW Bond Ave, CHH1 16th Floor, Portland, OR, 97239, United States, 1 503-418-3376, 1 503-494-6968; 3 College of Osteopathic Medicine, Western University of Health Sciences, Pomona, CA, United States; 4 School of Journalism and Communication, University of Oregon, Eugene, OR, United States; 5 Department of Dermatology, Warren Alpert Medical School of Brown University, Providence, RI, United States; 6 Department of Dermatology, University of Utah, Salt Lake City, UT, United States

**Keywords:** TikTok, social media, health information, misinformation, Global Quality Scale, GQS, dermatology, sunscreen, sun safety, sun protection factor, SPF

## Abstract

**Background:**

TikTok, with more than 2 billion users worldwide, has become an influential venue for health information, including dermatologic advice. However, concerns remain about the accuracy and impact of sunscreen-related content.

**Objective:**

This study aimed to assess the quality, accuracy, and themes of popular TikTok videos about sunscreen; evaluate associations with creator credentials and promotional content; and identify implications for public health.

**Methods:**

We conducted a cross-sectional content analysis of the 100 most-liked English-language TikTok videos generated by the search term “sunscreen.” Metadata, creator characteristics, Global Quality Score (GQS), accuracy, attitudes, promotional disclosures, and reference use were extracted using a structured codebook. Thematic and statistical analyses (ie, Pearson correlations, *χ*^2^, 2-tailed *t* tests, and ANOVA) were conducted, with significance defined as *P*<.05.

**Results:**

Of the top 100 videos, 74 (74%) expressed a positive attitude toward sunscreen, 35 (35%) were accurate, 57 (57%) were opinion based, and 6 (6%) were inaccurate. None of the videos cited references. GQS ratings were low: 40 (40%) videos were rated poor (score=1), 31 (31%) below average (score=2), and only 2 (2%) excellent (score=5). Promotional content appeared in 27 (27%) videos. Accuracy was negatively correlated with likes (*r*=–0.229; *P*=.02) and views (*r*=–0.242; *P*=.02), while GQS correlated positively with accuracy (*r*=0.270; *P*=.007) but not with engagement. Likes and views were strongly correlated (*r*=0.726; *P*<.001).

**Conclusions:**

Despite broadly positive sentiment toward sunscreen, misinformation and promotional bias are common in highly engaged TikTok videos, and user engagement is often unrelated to accuracy or educational quality. Dermatologists and public health experts must proactively engage on social platforms to counter misinformation and promote reliable skin health information.

## Introduction

Social media is an increasingly important source of health information, and TikTok, one of the world’s fastest-growing video-sharing platforms, has rapidly become a key venue for health messaging [1]. With its short-form video style and algorithmic feed, the platform shapes health behaviors, particularly among younger audiences. Within dermatology, TikTok content is both vast and highly visible, with dermatology-related hashtags collectively accumulating more than 18 billion views [2]. Sunscreen, a cornerstone of skin cancer prevention and photoaging mitigation, has emerged as a recurring theme across the platform, with *#sunscreen* alone garnering billions of views [3]. However, within the same feed that promotes sun protection, viral hashtags such as *#AntiSunscreen* circulate misinformation, propagating doubt about established guidance and exposing users considered vulnerable to confusion about one of the most important preventive health behaviors [4,5].

Existing research highlights the complexity of sunscreen discourse on TikTok (ByteDance Ltd). Haff et al [6] reported that much of dermatology content is low quality, with an average DISCERN score of 1.58 out of 5. Pediatric analyses similarly found that only 26% of daytime skincare regimens included sunscreen, despite frequent use of sun-sensitizing ingredients [7]. At the other extreme, misinformation has proliferated: Kearney et al [4] showed that 69% of videos under *sunscreen conspiracy* searches were antisunscreen, and Nikookam et al [5] emphasized the viral reach of the *#AntiSunscreen* movement. Even outside conspirative spaces, video quality is limited. Khan et al [3] found that the most popular sunscreen videos averaged a DISCERN score of 2.68 out of 5. In addition, promotional content is common: Ranpariya et al [8] showed that dermatology influencers frequently post sponsored material, raising questions about transparency and commercial influence.

Despite these insights, no study has systematically examined whether the most-engaged sunscreen content aligns with evidence-based dermatology guidance or how popularity favors accuracy. Our study addresses this gap through a systematic evaluation of the 100 most-liked *#sunscreen* TikTok videos, assessing accuracy, Global Quality Scale (GQS) score, sentiment, references, and promotional content and analyzing their relationship to engagement metrics.

## Methods

### Ethical Considerations

As this study relied exclusively on publicly available data without human participants, it was reviewed by the Oregon Health & Science University Institutional Review Board and deemed not to be human research (00027957).

### Study Design

This cross-sectional content analysis aimed to evaluate the quality, accuracy, and attitudes of popular sunscreen content on TikTok.

### Preliminary Screening and Search Strategy

To establish a search strategy, 2 reviewers (KD and JR-M) first conducted exploratory searches using targeted terms such as “toxic sunscreen,” “sunscreen risks,” and “sunscreen myths.” On the basis of these findings, we selected the broad search term “sunscreen,” limiting our final dataset to a single, neutral term to minimize search bias and ensure broad inclusion of relevant content. This approach enabled capturing a wide range of perspectives within a single organic search.

### Sample Selection

The final search was conducted on August 14, 2024, using newly created TikTok accounts to reduce algorithmic personalization. We applied TikTok’s “most-liked” filter and identified the top 106 most-liked videos returned by the search term “sunscreen.” For each video, we recorded metadata (URL, creator username, date posted, number of likes, views, and creator follower count) in Microsoft Excel (Microsoft Corporation) at the time of search.

### Inclusion and Exclusion Criteria

The top 100 most-liked videos were screened for inclusion. Eligible videos were required to (1) be in English or contain English subtitles and (2) be relevant to sunscreen use, safety, or recommendations. Duplicates and irrelevant videos were excluded. Videos ranked 101 to 106 were used exclusively for coder calibration and interrater reliability (IRR) testing. A sample size of 100 was selected to balance feasibility of detailed manual coding with representativeness. Although larger-scale TikTok analyses exist, such as the study by Steinke et al [9], our aim was to balance depth with representativeness.

### Variables and Coding Definitions

Videos were coded for the following variables:

- Content and creator characteristics included the date posted, creator username, number of likes, number of views, and follower count (recorded at the time of data collection).
- Content quality or usability was assessed using the 5-point GQS described by Bernard et al [10] (1=poor to 5=excellent); in our study, GQS anchors emphasized viewer-facing usefulness and clarity (refer to “Codebook Development and IRR” ).
- Accuracy was categorized as accurate, inaccurate, mixed, or opinion-based, according to concordance with the American Academy of Dermatology sunscreen guidelines [11].
- Reference use was coded based on presence or absence of identifiable sources such as peer-reviewed articles.
- Attitudes toward sunscreen were categorized as positive, negative, or neutral.
- Promotional content was identified by the presence of TikTok “Paid Partnership” labels, affiliate links, discount codes, or clearly disclosed sponsorships in captions or on-screen text.

### Codebook Development and IRR

Two reviewers (KD and JR-M) independently coded 6 calibration videos (videos 101-106) to refine the structured codebook (Multimedia Appendix 1) and assess IRR. IRR for categorical variables was evaluated using percent agreement, Scott pi, Cohen kappa, and Krippendorff alpha. Using the original 1‐5 GQS wording from prior literature [10,12-14], initial GQS agreement was 60% despite more than 85% agreement on other variables.

To improve consistency, coders received additional training and in-person calibration meetings, clarified the construct to reflect how lay viewers interpret scientific information, and iteratively revised scale anchors: 1=poor (largely missing information or unhelpful for viewer use), 2=below average (minimal content with major omissions), 3=moderate (some helpful information but incomplete or unclear for viewers), 4=good (covers main points understandably, though not comprehensive), and 5=excellent (clear, well structured, comprehensive, and highly useful).

Reviewers completed several rounds of independent recoding and consensus review over a few weeks. Disagreements were resolved by consensus using the written anchors and comparison to exemplar videos.

Calibration continued until IRR exceeded 0.90 across all variables and GQS reliability surpassed 0.75. After this, full extraction was conducted, with 1 reviewer coding all 100 videos and the second reviewer independently coding a blinded 10% (10/100) subset. Final IRR for this subset remained high (agreement=97.9%; Scott pi=0.954; Cohen kappa=0.954; Krippendorff alpha=0.956; Multimedia Appendix 2).

### Statistical Analysis

Descriptive statistics summarized video characteristics, themes, and creator demographics. Inferential tests included independent samples *t* tests and one-way ANOVA for comparing continuous variables across groups, Pearson correlations to examine relationships between engagement metrics and quality indicators (ie, accuracy and GQS), and *χ*^2^ tests for associations between categorical variables. Statistical significance was defined as *P*<.05. All analyses were conducted in Microsoft Excel and R (R Foundation for Statistical Computing). No videos were excluded due to missing data.

### Decile-Based Engagement Analysis

Because engagement metrics (ie, likes and views) were highly skewed, we performed a decile-based stratification. Videos were grouped by engagement deciles, and accuracy patterns were examined across strata to identify nonparametric trends that might have been obscured by extreme outliers.

## Results

### Descriptive Overview

Analysis of the 100 most-liked TikTok videos generated using the search term “sunscreen” revealed a mean like count of 227,671 (SD 351,902; range 26,700‐2,400,000) and a mean view count of 4,203,243 (SD 5,775,935; range 309,800‐34,300,000). The average number of followers per creator was 1,033,824 (SD 2,977,532; range 272‐18,100,000). Of the 100 content creators, only 18 (18%) identified as health care professionals, including 13 (13%) dermatologists, 2 (2%) medical aestheticians, 1 (1%) pharmacist, 1 (1%) esthetic nurse practitioner, and 1 (1%) cosmetic chemist. Ingredient mentions appeared in 13 (13%) videos, and toxicity concerns in 10 (10%) videos. Positive sunscreen attitudes were expressed in 74 (74%) videos.

Out of the 100 videos, 35 (35%) were classified as accurate, 6 (6%) as inaccurate, 2 (2%) as mixed, and 57 (57%) were opinion-based or not applicable for factual accuracy evaluation. Positive attitudes toward sunscreen were expressed in 74 (74%) videos, with the remainder split between 17 (17%) neutral and 9 (9%) negative sentiment videos.

Sunscreen product recommendations appeared in 85 (85%) videos, and 27 (27%) videos contained explicit promotional content such as affiliate links, brand sponsorships, or paid advertisements (Table 1).

**Table 1.** Descriptive characteristics of the top 100 most-liked TikTok videos related to sunscreen, including content accuracy, Global Quality Scores (GQS), thematic content, and creator background.

Characteristics		TikTok videos, n (%)
Attitude		
Positive		74 (74)
Negative		7 (7)
Neutral		9 (9)
Accuracy		
Opinion-based		57 (57)
Accurate		35 (35)
Inaccurate		6 (6)
Mixed		2 (2)
Used references		0 (0)
GQS		
1 (Poor quality)		40 (40)
2 (Below average)		31 (31)
3 (Moderate)		23 (23)
4 (Good)		4 (4)
5 (Excellent)		2 (2)
Content type		
Non-white skin–specific content (eg, white cast, blending, or sunscreen considerations for darker skin tones)		14 (14)
Sun protection factor		30 (30)
Sun safety education		31 (31)
Specific ingredients		13 (13)
Toxicity		10 (10)
Content creator background		
Board-certified dermatologist		13 (13)
Medical esthetician		2 (2)
Cosmetic chemist		1 (1)
Pharmacist		1 (1)
Nurse practitioner		1 (1)
Promotional content		
Creators with advertisements or promotion		27 (27)

### Engagement Metrics by Accuracy

Although inaccurate videos were fewer in number, they received disproportionately high engagement. On average, the 6 (6%) inaccurate videos received 2,141,683 (SD 2,235,616) views and 119,383 (SD 194,427) likes and the 35 (35%) accurate videos received 2,732,594 (SD 3,067,518) views and 135,711 (SD 131,773) likes.

A strong positive correlation was observed between the number of likes and views (*r*=0.726; *df*=98; *P*<.001). In contrast, accuracy was negatively correlated with both likes (*r*=–0.229; *df*=98; *P*=.02) and views (*r*=–0.242; *df*=98; *P*=.02), indicating that less accurate content may attract more user engagement. Refer to Table 2 for all Pearson correlation coefficients among key video characteristics.

**Table 2.** Correlation coefficients (Pearson *r*) among key variables in sunscreen-related TikTok videos.

Comparison	*r*	*P* value
Likes vs views	0.73^a^	<.001
Likes vs attitude	−0.19	.06
Likes vs promotional	−0.16	.11
Likes vs accuracy	−0.23	.02
Likes vs GQS^b^	0.12	.24
Views vs attitude	−0.15	.13
Views vs promotional	−0.04	.67
Views vs accuracy	−0.24	.02
Views vs GQS	0.19	.05
Attitude vs promotional	0.05	.63
Attitude vs accuracy	0.1	.32

a Statistically significant correlations.

b GQS: Global Quality Score.

**Figure 1.**
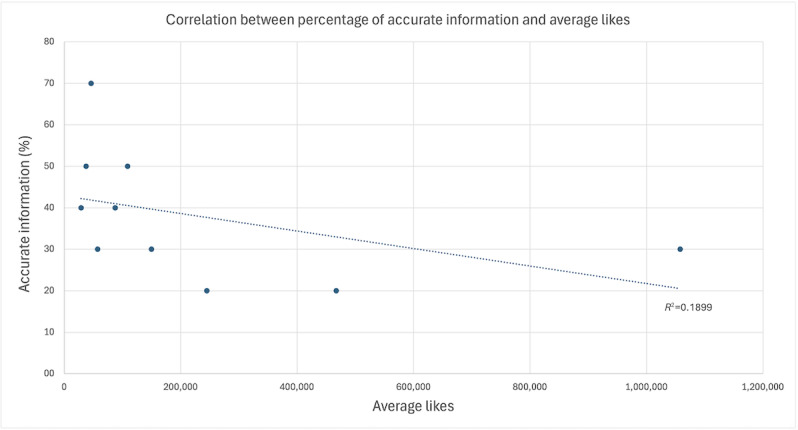
Relationship between accurate sunscreen content and views. The 100 most-liked videos were grouped into deciles based on their view counts. The percentage of videos within each decile that contain accurate information is shown on the y-axis, while the average views per decile are shown on the x-axis.

To account for the skewed distribution of engagement metrics, a decile-based analysis was conducted. This analysis revealed a strong, statistically significant negative correlation between accuracy and views (*r*=–0.720; *df*=8; *P*=.02) and a moderate, nonsignificant negative correlation between accuracy and likes (*r*=–0.440; *df*=8; *P*=.21). These findings suggest that less accurate videos may be algorithmically favored or more widely shared, resulting in greater visibility despite lower informational quality (Figures1  2).

**Figure 2.**
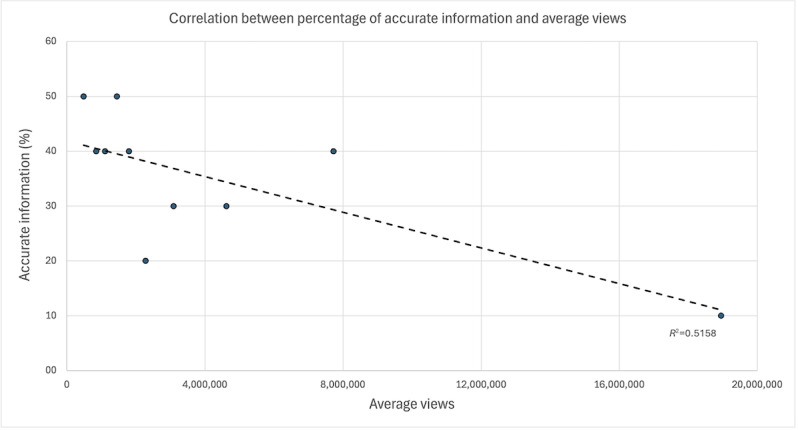
Relationship between accurate sunscreen content and likes. The 100 most-liked videos were grouped into deciles based on their like counts. The percentage of videos within each decile that contain accurate information is shown on the y-axis, while the average likes per decile are shown on the x-axis.

### GQS and Accuracy

Content quality was generally low across the top 100 most-liked sunscreen-related TikTok videos. The majority (71/100, 71%) of videos received GQS of 1 (poor quality) or 2 (below average), with only 2 (2%) scoring a 5 (excellent quality). GQS was positively correlated with accuracy (*r*=0.270; *df*=98; *P*=.007), indicating that videos deemed more accurate were also rated as higher quality. However, GQS showed no significant correlation with engagement metrics such as likes (*r*=0.118; *df*=98; *P*=.24) or views (*r*=0.193; *df*=98; *P*=.05), suggesting that content quality did not predict popularity on the platform. These findings reflect a disconnect between perceived usefulness and user engagement.

### Promotional Content

**Figure 3.**
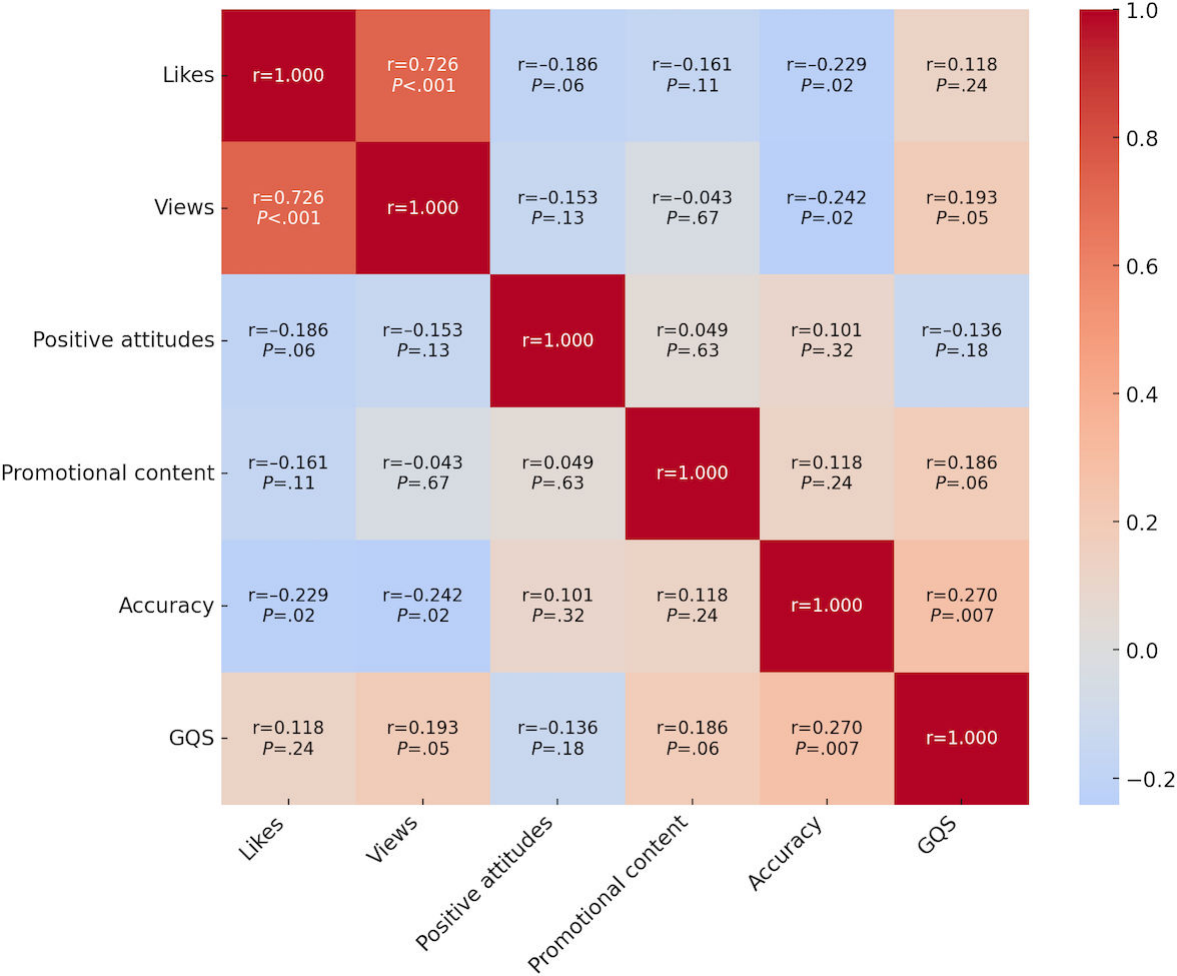
Heat map depicting Pearson correlations among key variables across the top 100 most-liked TikTok videos about sunscreen. Variables include Global Quality Score (GQS), accuracy, likes, views, promotional content, and positive attitudes. Warmer colors indicate positive correlations; cooler colors indicate negative correlations.

Promotional content was present in 27 (27%) of the top 100 most-liked sunscreen-related TikTok videos. However, the presence of promotional material was not significantly correlated with either likes (*r*=–0.161; *df*=98; *P*=.11) or views (*r*=–0.043; *df*=98; *P*=.67), suggesting that promotional status alone did not drive engagement. Similarly, promotional content showed only weak, nonsignificant correlations with GQS (*r*=0.186; *df*=98; *P*=.06) and accuracy (*r*=0.118; *df*=98; *P*=.24). These findings indicate that promotional content was common but not necessarily more engaging or accurate than nonpromotional content. Figure 3 visualizes these correlations across 6 content variables.

## Discussion

### Principal Findings

Our analysis of trending TikTok content on sunscreen revealed an average of 4,203,243 views and 227,671 likes per video, highlighting the platform’s massive reach. Among the 100 most-liked videos, 74 (74%) expressed a positive attitude toward sunscreen, 35 (35%) contained accurate information, and only 6 (6%) contained inaccurate content, per the American Academy of Dermatology guidelines. Notably, 57 (57%) videos were purely opinion-based. Our study is the first to demonstrate that sunscreen content on TikTok is predominantly positive but frequently lacks factual grounding or educational value. These findings expand on prior reports of variable quality in dermatology TikToks by specifically examining sunscreen content, an area of high public interest and misinformation.

### Comparison With Prior Work

Consistent with prior literature, we found that videos deemed accurate were rated significantly higher in quality, as measured by the GQS, aligning with established findings that content accuracy enhances educational utility [3-6]. However, more than half of the videos (57/100, 57%) were categorized as opinion-based and lacked references, reflecting a shift toward anecdotal rather than evidence-based guidance.

Promotional content was present in 27 (27%) videos but was not significantly associated with engagement metrics or content quality. It showed only weak, nonsignificant correlations with GQS (*r*=0.186; *df*=98; *P*=.06), accuracy (*r*=0.118; *df*=98; *P*=.24), likes (*r*=–0.161; *df*=98; *P*=.11), and views (*r*=–0.043; *df*=98; *P*=.67), suggesting that the presence of commercial messaging neither enhanced nor substantially degraded video quality or popularity. Although Ranpariya et al [8] documented the prevalence of promotional content among dermatology influencers, our analysis suggests that such content does not necessarily drive engagement or correlate with accuracy.

Despite comprising only 6 (6%) videos, inaccurate content attracted disproportionately high engagement, receiving a mean of 2,141,683 views and 119,383 likes, which was comparable to that of the 35 (35%) accurate videos (mean views 2,732,594; mean likes 135,711). A strong negative correlation was found between accuracy and views in decile-based analysis (*r*=–0.72; *df*=8; *P*=.02), and a moderate but nonsignificant negative correlation was found between accuracy and likes (*r*=–0.44; *df*=8; *P*=.21). This reinforces prior concerns that social media virality may be driven more by sensationalism or esthetics than by informational accuracy [11-13].

### Implications

The low overall GQS (mean 1.97) emphasizes the poor educational utility of the majority of sunscreen-related content on TikTok. Despite their reach, most videos failed to meet standards of quality, evidence, or clarity. Content with promotional framing was particularly vulnerable to low-quality scores, even when expressing positive sunscreen messaging. Given that accurate content did not consistently outperform anecdotal or promotional posts in engagement, our findings reinforce the need for dermatologists to adapt communication strategies to the realities of algorithm-driven platforms.

To address these gaps, we recommend that health care providers stay informed about dermatology trends on social media, discuss misinformation and content sources with patients, and engage on platforms such as TikTok to provide evidence-based perspectives.

Encouragingly, 18% (18/100) of the content creators in our dataset were health care providers, and 1 board-certified dermatologist had more than 18 million followers, demonstrating the potential impact medical professionals can have. To further promote quality content, we advocate for standardized disclosure of conflicts of interest, citation of sources, and increased collaboration with platform moderators.

### SkinMedia Initiative

Our team is committed to continuing this work through the SkinMedia series. This initiative aims to systematically track dermatologic misinformation and translate findings into publicly accessible content. This work directly addresses the gap identified in the current literature by moving beyond analysis to the development of interventions that amplify accurate dermatologic messaging. By partnering with dermatologists, educators, and digital platforms, we aim to promote scientifically grounded messaging, particularly for younger audiences. Future work will include the dissemination of open-access educational resources.

### Limitations

This study is limited by its cross-sectional design and the selection of only the 100 most-liked videos, which may not reflect less popular but potentially higher-quality content. The large percentage of promotional content (27/100, 27%) may also have skewed findings. IRR for most variables was high; however, agreement on GQS was lower (κ=0.712), consistent with its known subjectivity. Additionally, most content creators were nonhealth care professionals, which may explain the infrequent use of citations.

### Conclusions

This study was one of the first to systematically analyze the most-liked sunscreen videos on TikTok, demonstrating an abundance of opinion-based, low-quality content despite an overall positive stance toward sunscreen use. We urge dermatologists and other health professionals to expand their digital presence to ensure that younger populations have access to engaging, reliable, and evidence-based skin health information. Future efforts should explore collaborative strategies with platforms and influencers to amplify high-quality dermatology content and counteract misinformation.

## Supplementary material

10.2196/70010 Multimedia Appendix 1 Example of the codebook framework for video content extraction.

10.2196/70010 Multimedia Appendix 2 Interrater reliability for key coded variables in a 10% blinded subset of videos, assessed by 2 independent reviewers. Percent agreement, Scott pi, Cohen kappa, and Krippendorff alpha are reported. “Nan” indicates invariant responses where chance-adjusted metrics could not be calculated due to lack of variability in responses.

## Abbreviations

GQS: Global Quality Scale
IRR: interrater reliability
