# Supplementary material for: Perception, Quality, and Accuracy of Sunscreen Content on TikTok: SkinMedia Cross-Sectional Content Analysis
Source: JMIR Dermatol. 2025 Dec 1;8:e70010. doi: 10.2196/70010 (PMC12670045; doi:10.2196/70010)
Supplement: Multimedia Appendix 1 [file derma-v8-e70010-s001.docx]

| **Variable** | **Definition** | **Possible Values** | **Examples** |
| --- | --- | --- | --- |
| Username | Creator's TikTok handle | Free text | @skincarebyxx |
| Date Posted | Date the video was posted | MM/DD/YYYY | 11/12/2024 |
| Likes | Number of likes at the time of data extraction | Integer | 245,000 |
| Views | Number of views at the time of data extraction | Integer | 3,400,000 |
| Followers | The creator’s follower count at the time of data extraction | Integer | 1,200,000 |
| Recommendation | Mentions or promotes a specific sunscreen product or brand | Yes / No | “I love this EltaMD sunscreen” |
| Mentions Side Effects | Mentions negative outcomes of sunscreen use (e.g., acne, irritation) | Yes / No | “This sunscreen broke me out” |
| Financial Disclosure | Indicates paid sponsorship, affiliate links, or discount codes | Yes / No / Unsure | “Eligible for commission,” “Use my code” |
| Credential Type | Whether the creator identifies as a medical professional | Yes / No  If Yes, list profession | Yes – Board-certified dermatologist |
| Attitude | The overall message of the video about sunscreen | Positive / Negative / Neutral | Positive: “Always wear SPF” Negative: “Sunscreen is toxic” Neutral: No narration |
| Accuracy | Scientific accuracy of claims made | Accurate / Inaccurate / Mixed / N/A | Accurate: “SPF 30 blocks 97% of UVB” Inaccurate: “Sunscreen causes cancer” Mixed: “Sunscreen prevents burns but disrupts hormones” |
| Global Quality Score (GQS) | Educational usefulness of video content for general viewers | 1 – 5 | 1=not useful, 2=limited use, 3=somewhat useful, 4=useful, and 5=very useful |
| Scientific References | Mentions or links to credible sources (e.g., journal articles, gov sites) | Yes / No | Caption links to AAD guidelines or PubMed article |

**Supplemental Table 1.** Example of the Codebook Framework for video content extraction.
