# Supplementary material for: Perception, Quality, and Accuracy of Sunscreen Content on TikTok: SkinMedia Cross-Sectional Content Analysis
Source: JMIR Dermatol. 2025 Dec 1;8:e70010. doi: 10.2196/70010 (PMC12670045; doi:10.2196/70010)
Supplement: Multimedia Appendix 2 [file derma-v8-e70010-s002.docx]

| **Variable** | **Percent Agree-ment** | **Scott's Pi** | **Cohen's Kappa** | **Krippen-dorff's Alpha** | **N Agree-ments** | **N Cases** |
| --- | --- | --- | --- | --- | --- | --- |
| Attitude towards sunscreen (positive=1, negative=2, neutral=3, N/A=0) | 100.0 | 1.0 | 1.0 | 1.0 | 10.0 | 10 |
| Sunscreen product and/or usage recommendations (Y=1, N=0) | 100.0 | 1.0 | 1.0 | 1.0 | 10.0 | 10 |
| Discussed sunscreen types (chemical vs. mineral/physical) (Y=1, N=0) | 90.0 | 0.737 | 0.733 | 0.747 | 9.0 | 10 |
| References used (Y=1, N=0) | 100.0 | nan | nan | nan | 10.0 | 10 |
| Healthcare professional (Y=1, N=0) | 100.0 | 1.0 | 1.0 | 1.0 | 10.0 | 10 |
| Video discussed and/or creator had an ad, financial compensation, or promotion factor (Y=1, N=0) | 100.0 | 1.0 | 1.0 | 1.0 | 10.0 | 10 |
| Discussed skin of color and/or race/ethnicity in sunscreen use or sun-safety (Y=1, N=0) | 100.0 | nan | nan | nan | 10.0 | 10 |
| Discussed SPF (Y=1, N=0) | 100.0 | 1.0 | 1.0 | 1.0 | 10.0 | 10 |
| Sun safety education provided (skin cancer, sunburns, UV indices, etc.) (Y=1, N=0) | 100.0 | 1.0 | 1.0 | 1.0 | 10.0 | 10 |
| Sunscreen review (applied sunscreens, compared sunscreen products, white cast content) (Y=1, N=0) | 100.0 | 1.0 | 1.0 | 1.0 | 10.0 | 10 |
| Discussed sunscreen ingredients (Y=1, N=0) | 100.0 | 1.0 | 1.0 | 1.0 | 10.0 | 10 |
| Discussed sunscreen risks (toxicity, harm, etc.) (Y=1, N=0) | 100.0 | 1.0 | 1.0 | 1.0 | 10.0 | 10 |
| Information accuracy: (accurate=1, inaccurate=2, mixture=3, N/A=0) | 100.0 | 1.0 | 1.0 | 1.0 | 10.0 | 10 |
| GQS score (1=not useful, 2=limited use, 3=somewhat useful, 4=useful, and 5=very useful) | 80.0 | 0.712 | 0.714 | 0.727 | 8.0 | 10 |
| Average | 97.857 | 0.954 | 0.954 | 0.956 | 9.786 | 10 |

**Supplemental Table 2**. Interrater reliability for key coded variables in a 10% blinded subset of videos, assessed by two independent reviewers. Percent agreement, Scott’s Pi, Cohen’s Kappa, and Krippendorff’s Alpha are reported. “Nan” indicates invariant responses where chance-adjusted metrics could not be calculated due to lack of variability in responses.
